# Functional analysis of *GALT* variants found in classic galactosemia patients using a novel cell‐free translation method

**DOI:** 10.1002/jmd2.12037

**Published:** 2019-05-09

**Authors:** Daffodil M. Canson, Catherine Lynn T. Silao, Salvador Eugenio C. Caoili

**Affiliations:** ^1^ Institute of Human Genetics, National Institutes of Health University of the Philippines Manila Manila Philippines; ^2^ Department of Biochemistry and Molecular Biology, College of Medicine University of the Philippines Manila Manila Philippines

**Keywords:** classic galactosemia, galactose‐1‐phosphate uridylyltransferase, GALT deficiency, HeLa‐based cell‐free expression system, missense variants

## Abstract

Classic galactosemia is an autosomal recessive disorder caused by deleterious variants in the galactose‐1‐phosphate uridylyltransferase (*GALT*) gene. GALT enzyme deficiency leads to an increase in the levels of galactose and its metabolites in the blood causing neurodevelopmental and other clinical complications in affected individuals. Two *GALT* variants NM_000155.3:c.347T>C (p.Leu116Pro) and NM_000155.3:c.533T>G (p.Met178Arg) were previously detected in Filipino patients. Here, we determine their functional effects on the GALT enzyme through *in silico* analysis and a novel experimental approach using a HeLa‐based cell‐free protein expression system. Enzyme activity was not detected for the p.Leu116Pro protein variant, while only 4.5% of wild‐type activity was detected for the p.Met178Arg protein variant. Computational analysis of the variants revealed destabilizing structural effects and suggested protein misfolding as the potential mechanism of enzymological impairment. Biochemical and computational data support the classification of p.Leu116Pro and p.Met178Arg variants as pathogenic. Moreover, the protein expression method developed has utility for future studies of *GALT* variants.

**SYNOPSIS**

This research report provides computational and functional evidence for the pathogenicity of p.Leu116Pro and p.Met178Arg variants, and demonstrates the applicability of a HeLa‐based cell‐free expression system in identifying functionally deleterious *GALT* variants.

## 1 INTRODUCTION

Pathogenic variants in the galactose‐1‐phosphate uridylyltransferase (*GALT*) gene causing loss or severe reduction of GALT enzyme activity can lead to a disorder called classic galactosemia (OMIM #230400).^1^ Untreated infants affected with this inborn error of metabolism suffer from feeding problems, failure to thrive, hepatocellular damage, bleeding, and sepsis.^2^ GALT (EC 2.7.7.12), the second metabolic enzyme in the Leloir pathway of galactose metabolism, catalyzes the conversion of galactose‐1‐phosphate and UDP‐glucose to glucose‐1‐phosphate and UDP‐galactose, respectively.^3^ Deficiency of this enzyme results in the inability to metabolize galactose and the accumulation of galactose‐1‐phosphate, which is thought to be one of the most significant pathogenic factors in galactosemia.^4^

As of January 2019, over 330 *GALT* gene variants, mostly single base substitutions, have been recorded in the GALT Database (http://www.arup.utah.edu/database/GALT/GALT_welcome.php). Protein expression studies using yeast, bacterial, and mammalian cell‐based systems have been used to assess the effects of *GALT* variants.^5^, ^6^, ^7^, ^8^ However, a majority of the reported variants in databases lack allele‐specific functional data that demonstrate their effect on the GALT enzyme activity. This is an important gap in galactosemia research that needs to be addressed since the effect of variants on GALT function can vary in severity. While protein expression studies can directly determine the effect of variants on GALT function, molecular modeling techniques can provide significant insights into the mechanism of enzymological impairment. Therefore, we conducted functional analysis of recombinant GALT and used computational data to investigate the effects of two novel *GALT* variants. However, instead of the established expression systems employed in previous experiments, we used a new method using a cell‐free mammalian‐coupled transcription/translation system,^9^, ^10^ which is faster and more convenient.

Previously, mutational analysis of the exons and flanking intronic regions of the *GALT* gene in Filipino classic galactosemia patients revealed two novel variants, c.347T>C (p.Leu116Pro) and c.533T>G (p.Met178Arg),^11^ neither previously investigated functionally. Both are absent from the gnomAD population database (http://gnomad.broadinstitute.org/). One patient was homozygous for the p.Leu116Pro variant; no other sequence variants were detected in all 11 exons of the gene.^11^ A missense variant at the same codon, p.Leu116Ile, resulted in normal enzyme activity in protein expression studies but decreased GALT activity in patient erythrocytes.^6^, ^12^ Another missense variant at this amino acid position, p.Leu116Val, has been reported in the Human Gene Mutation Database (http://www.hgmd.cf.ac.uk/ac/index.php) though no functional analysis has yet been performed on this variant. The patient with the p.Met178Arg variant was observed as a compound heterozygote with p.Val168Leu,^11^ a variant previously found to abolish GALT activity.^13^ We thus assessed the functional effect of p.Leu116Pro and p.Met178Arg variants, and bioinformatically assessed the resulting amino acid substitutions for effects on the GALT enzyme, in order to confirm the pathogenicity of these two variants.

## 2 METHODS

### 2.1 Patients

The *GALT* variants studied were found separately in two patients clinically diagnosed with classic galactosemia confirmed by biochemical analyses. Clinical data were obtained from a review of the medical records (Table S1, adapted from Estrada et al^11^).

### 2.2 Web‐based tools

Pre‐computed data were obtained from the Galactosemia Proteins Database 2.0 (http://www.protein-variants.eu/galactosemia/), which uses the crystal structure of human GALT (PDB code 5IN3) in the analysis of variants.^14^ This database employs several tools to determine the effect of known variants on the functional and structural features of the three enzymes involved in the Leloir pathway. Additionally, the BeAtMuSiC v1.0 program was used in predicting the changes in binding affinity between subunits of the dimeric crystal structure.^15^ The functional effects of amino acid substitutions were also determined through PolyPhen‐2 and SIFT web servers.^16^, ^17^ Lastly, Phos3D^18^ was used to predict phosphorylation events on the human GALT structure since the cell‐free expression system used in this study is capable of this post‐translational modification.

### 2.3 Expression and purification of recombinant GALT enzyme

The detailed procedures of template preparation, protein expression and purification of recombinant GALT enzyme are provided as supplementary material. PCR‐based site‐directed mutagenesis^19^ using custom‐designed primers was performed to introduce the desired nucleotide changes, c.347T>C and c.533T>G, into wild‐type *GALT* cDNA that would be translated into p.Leu116Pro and p.Met178Arg variant proteins, respectively. A schematic diagram of the method and gel images for the PCR and ligation products are shown in supporting files (Figures S1 and S2). Bidirectional DNA sequencing confirmed the success of the mutagenesis experiment (Figure S3). The 1140‐bp wild‐type and mutant *GALT* cDNA fragments and elements from the pT7CFE1 vector were then used in the PCR assembly of IVT templates. A complete 1779‐bp PCR fragment as template for *in vitro* cell‐free protein expression has the following elements: T7 promoter, internal ribosome entry site (IRES) and Kozak sequence at the 5′ end, *GALT* cDNA, poly‐A tail at the 3′ end, and a 6XHis tag after the start codon for purification purposes (Figure S4).

The wild‐type GALT, p.Leu116Pro variant, and p.Met178Arg variant were expressed using the HeLa‐based cell‐free expression system, 1‐Step Human High‐Yield Mini IVT kit (Pierce Biotechnology/Thermo Scientific, Rockford, IL). The IVT templates were mixed with the HeLa lysate supplemented with proprietary accessory proteins. Protein expression took place for 8 hours at 30°C. To check for background GALT activity of the crude lysate, one reaction mixture without an IVT template was run together with the samples under identical conditions. This also served as a blank in GALT enzyme activity measurements. The 6XHis‐tagged protein products were isolated by affinity purification using Ni‐NTA magnetic agarose beads, and the size and quality of eluted protein was checked through SDS‐PAGE and native PAGE.

### 2.4 GALT enzyme activity measurement

Unless otherwise stated, reagents for GALT enzyme assay were obtained from Sigma, St. Louis, MO. UDP‐glucose was from Carbosynth Limited, Compton, Berkshire, UK, and MgCl_2_ was from Invitrogen/Life Technologies, Carlsbad, CA.

The assay was performed as per the method described by Tang et al^13^ with modification of the total reaction volume. The activity of wild‐type and variant GALT proteins were assayed in 20 μL glycine buffer (100 mM, pH 8.7) containing 5 mM MgCl_2_, 5 mM DTT, 0.8 mM NADP, 0.6 mM UDP‐glucose, 1.2 mM galactose‐1‐phosphate, 5 μM glucose‐1,6‐bisphosphate, 0.05 U glucose‐6‐phosphate dehydrogenase, and 0.05 U phosphoglucomutase. The reaction mixtures were incubated at 37°C for 30 minutes. The formation of NADPH was quantified by monitoring the change in absorbance of the reaction mixture at 340 nm using the UV‐Vis application of NanoDrop 2000 Spectrophotometer (Thermo Scientific, Wilmington, DE). The relationship between release of glucose‐1‐phosphate and increase in NADPH production was quantified using the Beer‐Lambert equation, and the molar extinction coefficient of NADPH (6220 M^−1^ cm^−1^) was used in the computation.

## 3 RESULTS

### 3.1 *In silico* analysis of GALT variants

The PolyPhen‐2 and SIFT algorithms predict that p.Leu116Pro and p.Met178Arg variants damage protein function. These predictions are supported by the structural data obtained from several *in silico* analysis tools (Table 1, Supplementary Tables S3 and S4). The Galactosemia Proteins Database 2.0 (http://www.protein-variants.eu/galactosemia/) predicts both p.Leu116Pro and p.Met178Arg protein variants to be less stable than the wild‐type GALT enzyme. Leu116 and Met178 are highly conserved residues (Table 1); thus, they are likely to be crucial in maintaining the structural integrity of the GALT protein.

**Table 1:** Results of *in silico* structural analysis of p.Leu116Pro and p.Met178Arg GALT variants

	p.Leu116Pro		p.Met178Arg	
Structural features^a^	Chain A	Chain B	Chain A	Chain B
Secondary structure	+	+	+	−
Solvent accessibility	−	−	−	−
Intrachain interactions	+	+	+	+
Interchain interactions	+	+	+	+
Ligand interactions	−	−	−	−
H‐bonds^b^	+	+	+	+
Salt bridges^b^	−	−	−	+
Hydrophobic interactions	+	+	+	+
Predicted stability	Less stable	Less stable	Less stable	Less stable
Subunit binding affinity	Decreased		Slightly increased	
Wild‐type conservation score^c^	8		9	

+, affected; −, not affected.

a All results were obtained from the Galactosemia Proteins Database 2.0 except for subunit binding affinity, which was predicted using the BeAtMuSiC v1.0 program.

b Intrachain interactions.

c The score can vary between 0 and 10; the higher the score, the higher the conservation of the residue.

**Figure 1:**
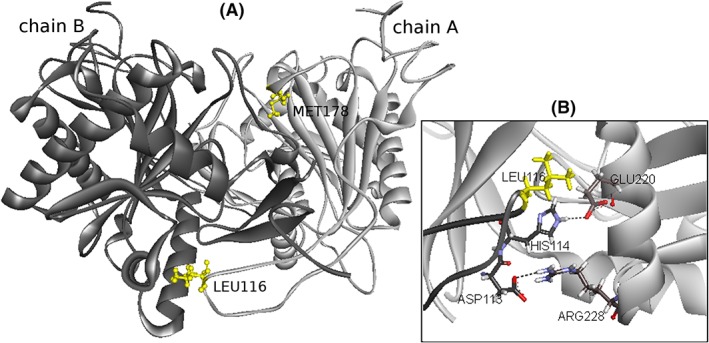
The structure of human GALT enzyme showing the affected residues. (A) Schematic view of the location of mutated amino acid residues in the dimer interface. The two subunits are shown in light gray (chain A) and dark gray (chain B). Affected residues are colored yellow and represented in ball‐and‐stick mode. (B) Leu116 is located close to the two salt bridges, Asp113^B^‐Arg228^A^ and His114^B^‐Glu220^A^. Redrawn from PDB file 5IN3 using Discovery Studio Visualizer 3.5 (Accelrys Software, Inc., San Diego, CA)

Figure 1A shows the schematic view of the location of mutated amino acid residues in the dimer interface. According to the BeAtMuSiC v1.0 program, the p.Leu116Pro variant decreases the binding affinity (ΔΔG_Bind_ = 5.95 kcal/mol) while the p.Met178Arg variant slightly increases the binding affinity (ΔΔG_Bind_ = −0.22 kcal/mol) between subunits. The p.Leu116Pro variant has a more deleterious effect on the affinity of the two subunits as reflected by the larger change in binding free energy. On the other hand, the p.Met178Arg variant may alter protein flexibility due to the slight increase in affinity as indicated by the negative sign of ΔΔG_Bind_. These results agree with the data obtained from the Galactosemia Proteins Database 2.0: the widening of interchain distance of residue 116 and narrowing of interchain distance of residue 178 (Table S3). Both are also predicted to lead to loss of interchain hydrophobic contacts; the p.Leu116Pro variant causes greater loss of hydrophobic interactions between subunits than the p.Met178Arg variant (Table S4). Moreover, the severe effect of p.Leu116Pro, that is, non‐detectable enzyme activity (see below), can be explained by its proximity to the salt bridges at the end of the dimerization loop (Figure 1B). The conformational change resulting from the amino acid substitution may indirectly affect these salt bridges and thus potentially impair the correct *in vivo* dimerization, meaning the variant is able to form dimers but the residue orientations may be affected.

### 3.2 GALT purification and enzyme activity measurement

SDS‐PAGE and native PAGE results show high‐purity recombinant GALT proteins after affinity purification (Figure S5). Native PAGE provided additional information on the effect of the variants on protein dimerization, a crucial feature of an active GALT enzyme. All three purified proteins, wild‐type (WT), p.Leu116Pro, and p.Met178Arg, exhibited similar single‐band profiles, suggesting that the two missense variants did not prevent the formation of the dimer as evidenced by the absence of monomeric subunits (44.2 kDa).

**Figure 2:**
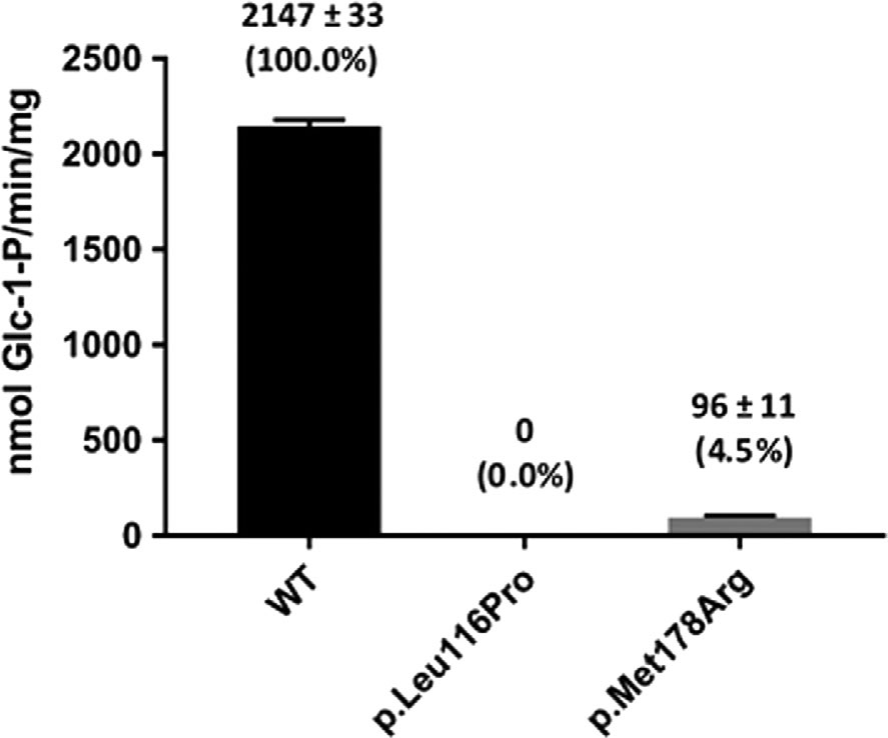
Enzymatic activity measurement of purified wild‐type and variant GALT proteins. 100 ng of purified protein was tested in a total volume of 20 μL glycine buffer. The activity values were generated from three separate experiments. The mean and SD are indicated above the bars. Values in parentheses reflect the % of wild type activity

Figure 2 shows the enzyme activities of the p.Leu116Pro and p.Met178Arg variants compared with wild‐type GALT. Both variants impaired the function of the GALT enzyme. The p.Leu116Pro variant had no detectable activity, while the p.Met178Arg variant exhibited only 4.5% of the activity of the wild‐type GALT.

## 4 DISCUSSION

The p.Leu116Pro and p.Met178Arg variants are predicted to destabilize the GALT enzyme by altering the secondary structure, H‐bond and hydrophobic interactions networks, as well as dimer flexibility in the case of p.Met178Arg. Decreased monomer stability suggests that these variants may cause protein misfolding, a common molecular mechanism of GALT deficiency in patients.^20^ Their mechanism of enzymological impairment may be similar to that of pathogenic variants p.Phe194Leu (exhibiting 12% of wild‐type GALT activity) and p.Arg333Gly (<1% of wild‐type activity), both of which are located at the dimer interface, not directly involved in substrate binding, but resulted in protein variants with reduced ability to bind substrates.^20^ Similarly, the p.Leu116Pro and p.Met178Arg protein variants are able to form dimers despite amino acid substitutions at the dimer interface, but are predicted to be less stable than the wild‐type protein; these two missense variants likely impair the correct *in vivo* dimerization of the protein and affect the orientation of residues at the active site.

This study has shown that the p.Leu116Pro and p.Met178Arg variants severely affect the function of the GALT enzyme. In the case of p.Met178Arg, it could not be ascertained whether the observed residual activity (4.5% of wild‐type activity) was due to endogenous GALT although this is unlikely since residual activity was not observed for the p.Leu116Pro variant. Whether the 4.5% activity has clinical implications remains to be seen since this is the first application of the method; more GALT variants need to be tested to assess the clinical relevance of the levels of residual activity in this system. It can be noted, however, that the patient harboring the p.Met178Arg allele appears to have better clinical presentation compared to the p.Leu116Pro homozygote (Table S1).

The presence of endogenous GALT as a limitation has been considered by previous research that used COS and 293 cells, where certain missense variants exhibited GALT activities that were significantly different from the activities derived from patient erythrocytes or lymphoblasts carrying those same variants.^6^, ^8^ For example, the classic variant allele p.Q188R exhibited 10% of wild‐type activity in the COS cell transfection system but showed no detectable activity in patient lymphoblasts that were homozygous for this variant.^8^ Nevertheless, this study is the first to demonstrate that a HeLa‐based cell‐free system can be used to express variant GALT enzymes to rapidly confirm the nature of sequence variants. This expression system is also capable of post‐translational modifications such as phosphorylation. This is a significant feature because the GALT protein is predicted to be phosphorylated at Ser108, Ser205, Thr268, and Thr350 based on the Phos3D algorithm.^18^ Previous functional studies of *GALT* gene variants used heterologous expression systems such as COS cells^8^ and 293 cells,^6^ yeast,^5^ or *E. coli*.^7^ Although heterologous expression certainly has its own merits such as the absence of endogenous GALT in previously developed yeast and bacterial systems, it is generally expensive and labor‐intensive. Therefore, our *in vitro* protein expression method using cell‐free expression system provides a good alternative for convenient and faster screening to identify functionally deleterious variants including those that might affect phosphorylation.

## 5 CONCLUSION

*In silico* analysis predicted that the p.Leu116Pro and p.Met178Arg variants are damaging to function via destabilizing structural effects on the human GALT protein. *in vitro* protein expression and GALT enzyme assay data also provided evidence of their effect on protein function. Together with the clinical features of patients first identified to carry these variants, the biochemical and computational data from this study strongly support the conclusion that p.Leu116Pro and p.Met178Arg are disease‐causing variants. Additionally, this study demonstrated the utility of a HeLa‐based cell‐free expression system to screen for *GALT* variants encoding proteins with abrogated function.

## CONFLICT OF INTEREST

The authors declare that they have no competing interests.

## INFORMED CONSENT

This study was reviewed and approved by the University of the Philippines Manila Research Ethics Board (Project code: UPMREB‐2013‐028‐P1). Informed consent was waived because biological samples from individuals were not used. The starting material (*GALT* cDNA) is commercially available so extracting blood from patients was not necessary.

## ETHICAL APPROVAL

This article does not contain any studies with animal subjects performed by any of the authors.

## AUTHOR CONTRIBUTIONS

Daffodil M. Canson, the corresponding author, conceived and designed the study, did the molecular analysis, obtained computational data, and wrote the manuscript with input from other authors. Catherine Lynn T. Silao and Salvador Eugenio C. Caoili supervised the study and contributed to drafting and critically revising the article. All authors read and approved the final manuscript.

## Supporting information


**Figure S1** Schematic diagram of single‐codon mutagenesis using *Sap*I restriction enzyme. The diagram illustrates the generation of a T>C substitution mutation. Two separate PCR fragments are produced from the template *GALT* cDNA. The two mutagenic primers contain mutated codon sequences that are complementary to each other and adjacent to the *Sap*I recognition site (5′‐GCTCTTCN). After *Sap*I digestion, two fragments are ligated together to generate the mutated full‐length *GALT* cDNA

**Figure S2** Gel image of PCR and ligation products in site‐directed mutagenesis. (a) Two fragments were produced from the template human *GALT* cDNA for each variant, p.L116P (116A and 116B) and p.M178R (178A and 178B). The ends of these paired fragments contain the mutated codon sequences that are complementary to each other and adjacent to the *Sap*I recognition site. *Lane 1*‐1 kb Plus DNA ladder, *lanes 2 and 3*—116A and 116B, *lanes 4 and 5*—178A and 178B. (b) After *Sap*I digestion, the two fragments were ligated together to generate the mutated full‐length *GALT* cDNA (p.L116P: 116A + 116B, p.M178R: 178A + 178B). *Lane 1*—100 bp DNA ladder, *lane 2*—control fragments without T4 DNA ligase, *lane 3*‐ ligation reaction with T4 DNA ligase

**Figure S3** Verification of mutation by sequence analysis. The introduced sequence changes, that is, c.347T>C (p.L116P) and c.533T>G (p.M178R), were confirmed through bidirectional DNA sequencing through capillary electrophoresis

**Figure S4** Gel image of T7‐IRES‐Kozak (T7IK) fragment and complete IVT template. The T7IK and full‐length *GALT* cDNA fragments were joined together through extension PCR to generate the complete IVT template

**Figure S5** SDS‐PAGE and native PAGE gel images of purified GALT proteins. SDS‐PAGE showed the expected size (44.2 kDa) of the recombinant GALT protein. Native PAGE demonstrated that all three GALT proteins were able to dimerize. *Lane 1*—WT, *lane 2*—p.Leu116Pro, *lane 3*—p.Met178Arg

**Table S1** Clinicodemographic data of two patients with Classic Galactosemia (adapted from^11^ Estrada et al 2013)

**Table S2** Primers for PCR amplification and sequencing

**Table S3** Interchain distances of affected residues in the GALT structure (adapted from the GALT Proteins Database 2.0)

**Table S4** Interchain hydrophobic contacts of affected residues in the GALT structure (adapted from the GALT Proteins Database 2.0)
