# Supplementary figures and images for: Functional analysis of GALT variants found in classic galactosemia patients using a novel cell‐free translation method
Source: JIMD Rep. 2019 May 9;48(1):60–6. doi: 10.1002/jmd2.12037 (PMC6606980; doi:10.1002/jmd2.12037)

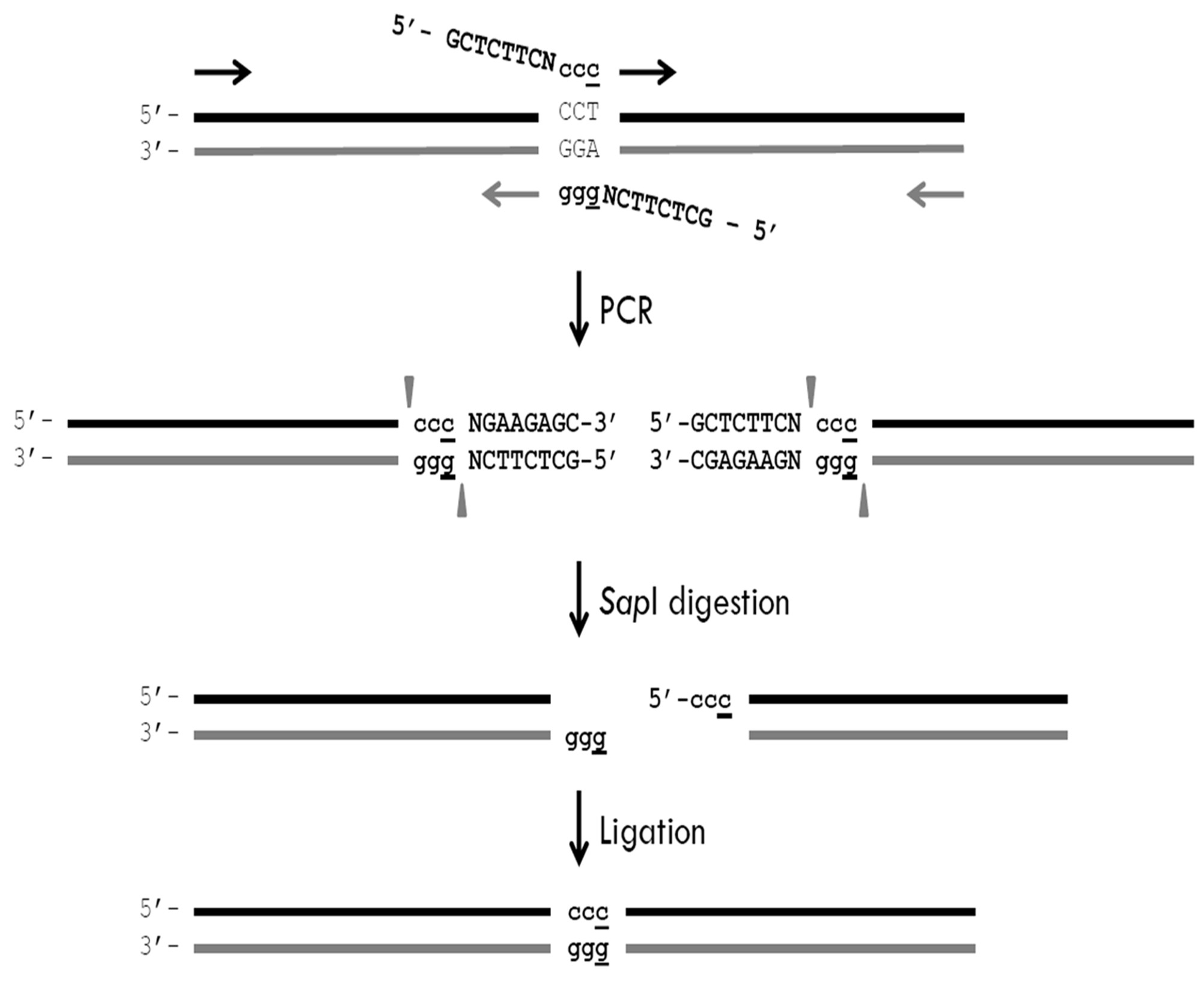

Supplement: Supplementary file 1 — Figure S1 Schematic diagram of single‐codon mutagenesis using SapI restriction enzyme. The diagram illustrates the generation of a T>C substitution mutation. Two separate PCR fragments are produced from the template GALT cDNA. The two mutagenic primers contain mutated codon sequences that are complementary to each other and adjacent to the SapI recognition site (5′‐GCTCTTCN). After SapI digestion, two fragments are ligated together to generate the mutated full‐length GALT cDNA [file JMD2-48-60-s001.jpeg]

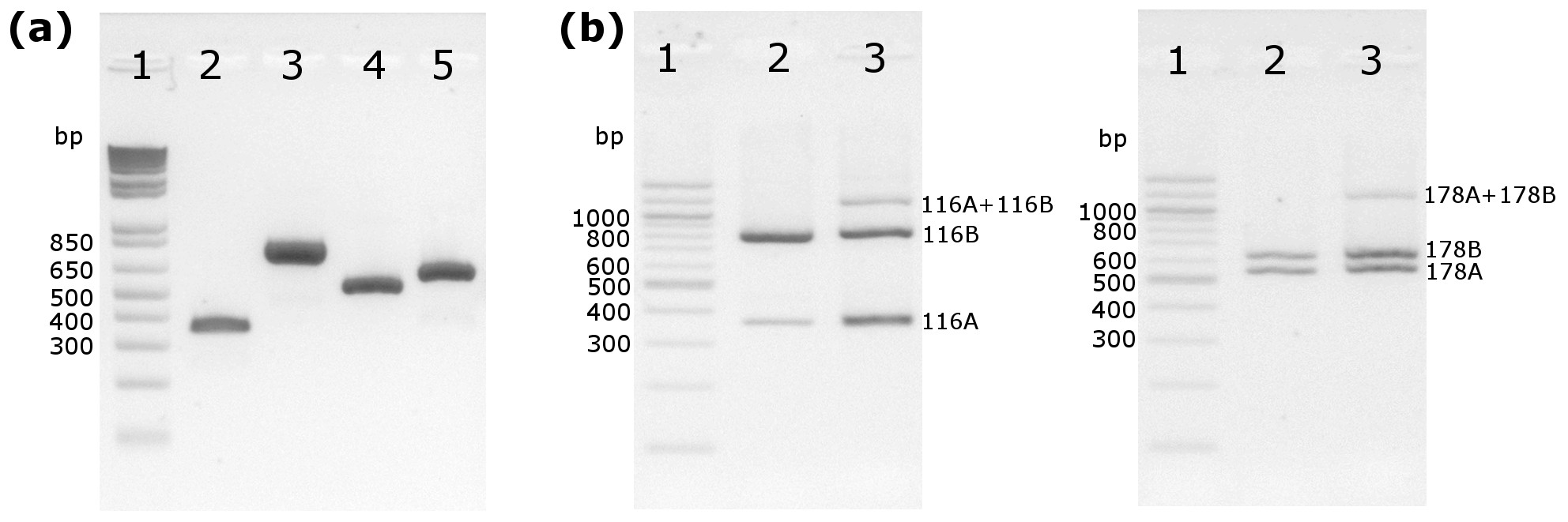

Supplement: Supplementary file 2 — Figure S2 Gel image of PCR and ligation products in site‐directed mutagenesis. (a) Two fragments were produced from the template human GALT cDNA for each variant, p.L116P (116A and 116B) and p.M178R (178A and 178B). The ends of these paired fragments contain the mutated codon sequences that are complementary to each other and adjacent to the SapI recognition site. Lane 1‐1 kb Plus DNA ladder, lanes 2 and 3—116A and 116B, lanes 4 and 5—178A and 178B. (b) After SapI digestion, the two fragments were ligated together to generate the mutated full‐length GALT cDNA (p.L116P: 116A + 116B, p.M178R: 178A + 178B). Lane 1—100 bp DNA ladder, lane 2—control fragments without T4 DNA ligase, lane 3‐ ligation reaction with T4 DNA ligase [file JMD2-48-60-s002.jpeg]

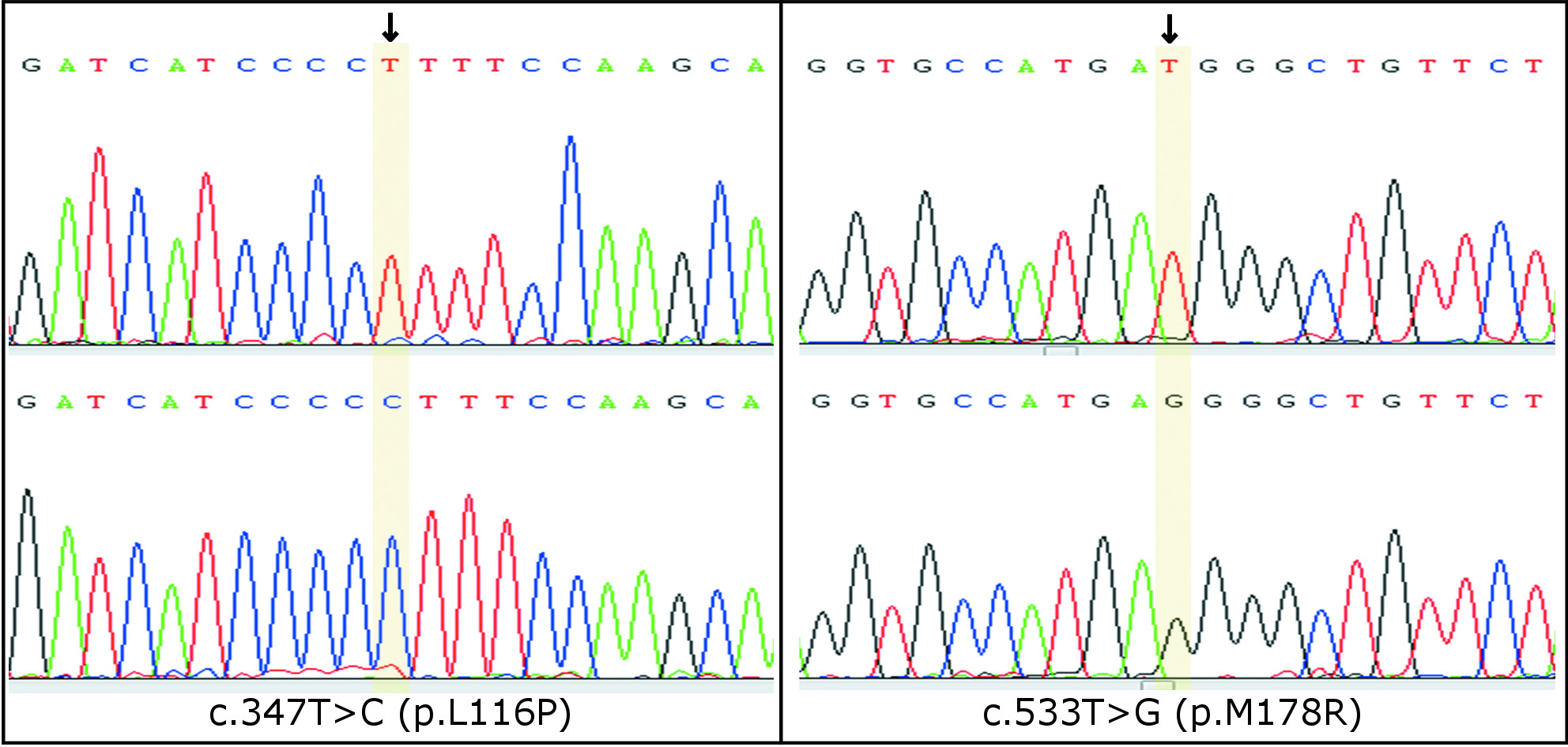

Supplement: Supplementary file 3 — Figure S3 Verification of mutation by sequence analysis. The introduced sequence changes, that is, c.347T>C (p.L116P) and c.533T>G (p.M178R), were confirmed through bidirectional DNA sequencing through capillary electrophoresis [file JMD2-48-60-s003.jpeg]

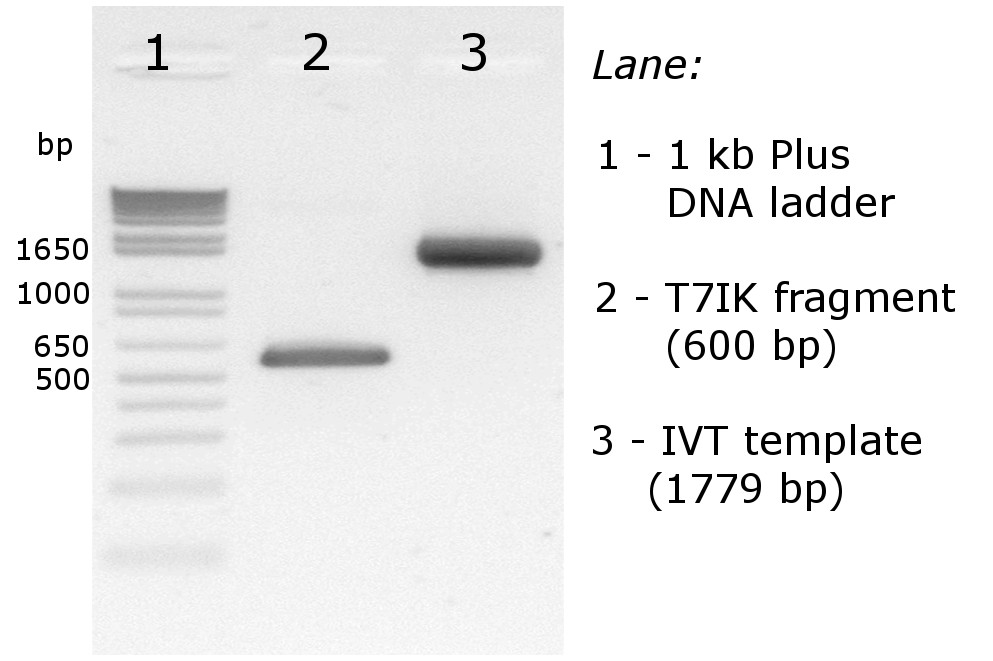

Supplement: Supplementary file 4 — Figure S4 Gel image of T7‐IRES‐Kozak (T7IK) fragment and complete IVT template. The T7IK and full‐length GALT cDNA fragments were joined together through extension PCR to generate the complete IVT template [file JMD2-48-60-s004.jpeg]

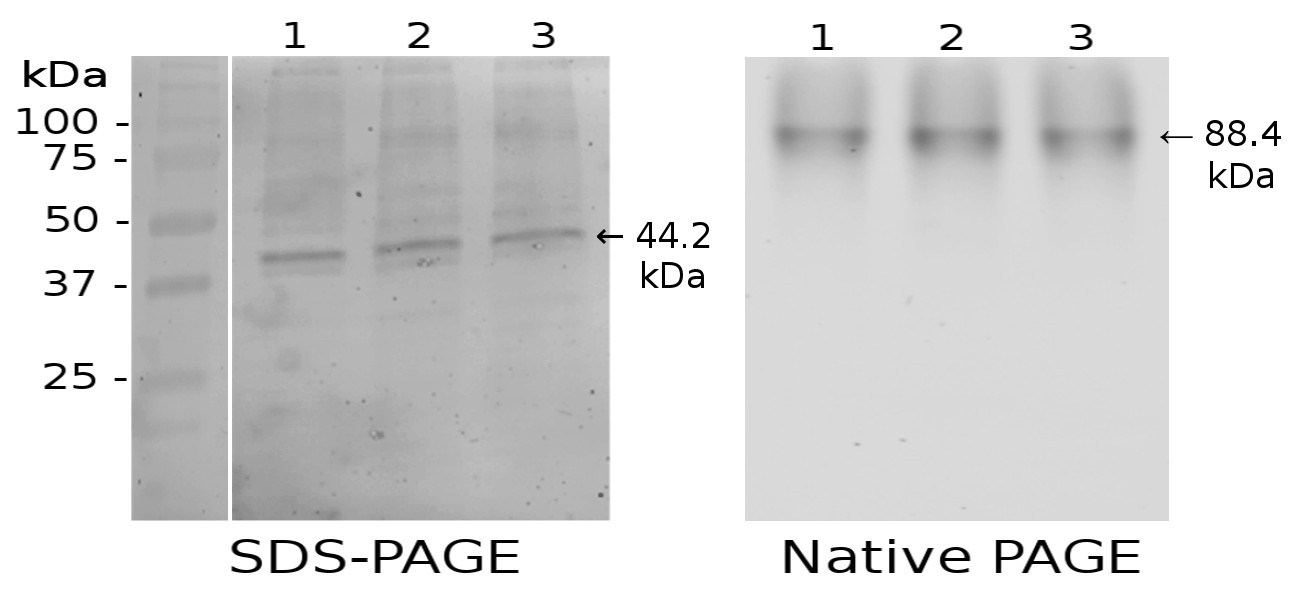

Supplement: Supplementary file 5 — Figure S5 SDS‐PAGE and native PAGE gel images of purified GALT proteins. SDS‐PAGE showed the expected size (44.2 kDa) of the recombinant GALT protein. Native PAGE demonstrated that all three GALT proteins were able to dimerize. Lane 1—WT, lane 2—p.Leu116Pro, lane 3—p.Met178Arg [file JMD2-48-60-s005.jpg]
