# Supplementary material for: Functional analysis of GALT variants found in classic galactosemia patients using a novel cell‐free translation method
Source: JIMD Rep. 2019 May 9;48(1):60–6. doi: 10.1002/jmd2.12037 (PMC6606980; doi:10.1002/jmd2.12037)
Supplement: Supplementary file 6 — Table S1 Clinicodemographic data of two patients with Classic Galactosemia (adapted from11 Estrada et al 2013) [file JMD2-48-60-s006.docx]

**Table S1. Clinicodemographic data of two patients with Classic Galactosemia** (adapted from Estrada et al 2013)

|  | | | **Patient 1**  **(male)** | **Patient 2**  **(male)** |
| --- | --- | --- | --- | --- |
| **Clinical information** | **Gestational age (weeks)** | | 38 | 36 |
|  | **Birthweight (g)** | | 3232 | 3000 |
|  | **Consanguinity** | | + | - |
|  | **Onset age** | | 3 days old | 4 days old |
|  | **Clinical presentation** | On initial consult | Jaundice and sepsis occurred in the first two weeks.  Hepatomegaly was noted at 1 month.  Treatment with gal-free diet started after diagnosis. | At day 1, fed milk formula and was noted to have icteric sclerae, soft, non-watery stools.  At 4 days, jaundice, diarrhea, seizures, irritability and poor suck occurred. Hepatomegaly and sepsis were noted at 7 days.  Treatment with gal-free diet started after diagnosis. |
|  |  | At follow up | At 2 years and 6 months, development noted to be normal. Neither liver abnormality nor cataracts were observed.  On regular follow up.  At 11 y.o., no cataracts. Grade 6 average student. Normal development. No liver abnormality | At 2 mos, history of poor suck, watery stools on breastmilk feeds.  At 11 months, liver function was normal.  At 1 y.o., elevated liver enzymes and normal liver on ultrasound.  At 4 y.o., noted to have significant speech delay.  At 5 y.o., with global developmental delay (intellectual disability with difficulty in the cognitive domain). Presently attends special education and speech therapy.  On regular follow up. At present, no cataracts. No hepatomegaly. |
| **Biochemical findings** | **GALT activity in erythrocytes** | From whole blood | None | None |
|  | **GALT activity in erythrocytes from dried blood spots** | On newborn screening | Not detectable | Not detectable |
|  |  | At follow up | Not detectable | Not detectable |
|  | **Total Galactose (Gal + Gal-1-P) level** | Initial level | 1 mmol/L | 0.2 mmol/L |
|  |  | At follow up | 0.2 mmol/L | 0.1-0.2 mmol/L |
| **Genotype** | | | p.Val168Leu/ p.Met178Arg | p.Leu116Pro/ p.Leu116Pro |
