# Supplementary material for: Functional analysis of GALT variants found in classic galactosemia patients using a novel cell‐free translation method
Source: JIMD Rep. 2019 May 9;48(1):60–6. doi: 10.1002/jmd2.12037 (PMC6606980; doi:10.1002/jmd2.12037)
Supplement: Supplementary file 7 — Table S2 Primers for PCR amplification and sequencing [file JMD2-48-60-s007.docx]

**Table S2.** Primers for PCR amplification and sequencing

| **Primer** | **Sequence (5’ – 3’)** | **Use** |
| --- | --- | --- |
| GALT-aF | ATG TCG CGC AGT GGA ACC | Sequencing, site-directed mutagenesis |
| GALT-aR | TCA CGC TGG GCA ATA TCT GG | Sequencing |
| GALT-bF | GAT ATT GCC CAG CGT GAG GA | Sequencing |
| GALT-bR | CTA GGC GAT GGT TGC TGT CTC | Sequencing, site-directed mutagenesis |
| Upstream T7 Forward^†^ | AAC GAC GGC CAG TGA ATT GTA ATA | Isolation of T7-IRES-Kozak fragment |
| IRES Reverse^†^ | ATG GGT GGT GGC CAT ATT ATC ATC |  |
| cGALT-F | GAT GAT AAT ATG GCC ACC ACC CAT ATG CAT CAT CAC CAC CAC CAT TCG CGC AGT GGA ACC GAT | PCR Step 1, generation of template for *in vitro* translation |
| cGALT-R | TTT TTT TTT TTT TTT TTT TTT CTA GGC GAT GGT TGC TGT CTC |  |
| SapI-116F | AAG CTC TTC CCC CTT TCC AAG CAA AGT CTG | Site-directed mutagenesis, generation of p.L116P mutant cDNA |
| SapI-116R | AAG CTC TTC AGG GGG ATG ATC ACT GGG TC |  |
| SapI-178F | AAG CTC TTC TGA GGG GCT GTT CTA ACC C | Site-directed mutagenesis, generation of p.M178R mutant cDNA |
| SapI-178R | AAG CTC TTC CCT CAT GGC ACC TTT GTT TTC |  |
| ^†^Sequence copied from Thermo Scientific TECH TIP #72 (Pierce Biotechnology 2011) | | |
