# Supplementary material for: Functional analysis of GALT variants found in classic galactosemia patients using a novel cell‐free translation method
Source: JIMD Rep. 2019 May 9;48(1):60–6. doi: 10.1002/jmd2.12037 (PMC6606980; doi:10.1002/jmd2.12037)
Supplement: Supplementary file 8 — Table S3 Interchain distances of affected residues in the GALT structure (adapted from the GALT Proteins Database 2.0) [file JMD2-48-60-s008.docx]

**Table S3.** Interchain distances of affected residues in the GALT structure (adapted from the GALT Proteins Database 2.0)

| **Residue: Chain** | **Wild-type** | | | **Variant** | | |
| --- | --- | --- | --- | --- | --- | --- |
|  | **Atom 1** | **Atom 2** | **Value (Å)** | **Atom 1** | **Atom 2** | **Value (Å)** |
| **116:A** | LEU A 116 (CD1) | GLN B 224 (OE1) | 2.916 | PRO A 116 (O) | PRO B 36 (CG) | 3.271 |
| **116:B** | LEU B 116 (CD2) | TYR A 209 (OH) | 2.805 | PRO B 116 (CG) | TYR A 209 (CE2) | 3.701 |
| **178:A** | MET A 178 (CE) | VAL B 337 (CG2) | 2.950 | ARG A 178 (CZ) | VAL B 337 (CG2) | 2.713 |
| **178:B** | MET B 178 (O) | VAL A 337 (CG2) | 3.166 | ARG B 178 (O) | VAL A 337 (CG2) | 3.147 |
