# Supplementary material for: Functional analysis of GALT variants found in classic galactosemia patients using a novel cell‐free translation method
Source: JIMD Rep. 2019 May 9;48(1):60–6. doi: 10.1002/jmd2.12037 (PMC6606980; doi:10.1002/jmd2.12037)
Supplement: Supplementary file 9 — Table S4 Interchain hydrophobic contacts of affected residues in the GALT structure (adapted from the GALT Proteins Database 2.0) [file JMD2-48-60-s009.docx]

**Table S4.** Interchain hydrophobic contacts of affected residues in the GALT structure (adapted from the GALT Proteins Database 2.0)

| **Residue: Chain** | **Wild-type** | | | **Variant** | | |
| --- | --- | --- | --- | --- | --- | --- |
|  | **Atom 1** | **Atom 2** | **Type^†^** | **Atom 1** | **Atom 2** | **Type^†^** |
| **116:A** | PRO B 36 (CG) | LEU A 116 (O) | S→M | PRO B 36 (CG) | PRO A 116 (CA) | S→M |
|  | TYR B 209 (CE1) | LEU A 116 (CD2) | S→S | PRO B 36 (CG) | PRO A 116 (CB) | S→S |
|  | LEU B 217 (CD2) | LEU A 116 (CD2) | S→S |  |  |  |
|  | LEU B 217 (O) | LEU A 116 (CD1) | M→S |  |  |  |
|  | GLU B 220 (C) | LEU A 116 (CD1) | M→S |  |  |  |
|  | GLU B 220 (CB) | LEU A 116 (CD1) | S→S |  |  |  |
|  | TYR B 221 (CA) | LEU A 116 (CD1) | M→S |  |  |  |
|  | TYR B 221 (CB) | LEU A 116 (CD1) | S→S |  |  |  |
|  | TYR B 221 (N) | LEU A 116 (CD1) | M→S |  |  |  |
|  | GLN B 224 (CD) | LEU A 116 (CD1) | S→S |  |  |  |
|  | GLN B 224 (OE1) | LEU A 116 (CD1) | S→S |  |  |  |
|  | GLN B 224 (OE1) | LEU A 116 (CG) | S→S |  |  |  |
|  | TRP B 246 (CZ3) | LEU A 116 (O) | S→M |  |  |  |
| **116:B** | LEU B 116 (CD1) | LEU A 217 (CD2) | S→S | PRO B 116 (CB) | PRO A 36 (CG) | S→S |
|  | LEU B 116 (CD1) | LEU A 217 (CG) | S→S | PRO B 116 (CD) | TYR A 209 (CE2) | S→S |
|  | LEU B 116 (CD2) | TYR A 209 (CE2) | S→S | PRO B 116 (CG) | TYR A 209 (CE2) | S→S |
|  | LEU B 116 (CD2) | TYR A 209 (CZ) | S→S |  |  |  |
|  | LEU B 116 (CD2) | TYR A 209 (OH) | S→S |  |  |  |
|  | LEU B 116 (CG) | TYR A 209 (OH) | S→S |  |  |  |
| **178:A** | PHE B 335 (CB) | MET A 178 (O) | S→M | VAL B 337 (CB) | ARG A 178 (CZ) | S→S |
|  | VAL B 337 (CB) | MET A 178 (O) | S→M | VAL B 337 (CG1) | ARG A 178 (CZ) | S→S |
|  | VAL B 337 (CG1) | MET A 178 (O) | S→M | VAL B 337 (CG2) | ARG A 178 (CZ) | S→S |
|  | VAL B 337 (CG2) | MET A 178 (CE) | S→S |  |  |  |
| **178:B** | MET B 178 (CE) | VAL A 337 (CG1) | S→S | Not involved | | |
|  | MET B 178 (O) | VAL A 337 (CB) | M→S |  |  |  |
|  | MET B 178 (O) | VAL A 337 (CG1) | M→S |  |  |  |
|  | MET B 178 (O) | VAL A 337 (CG2) | M→S |  |  |  |

^†^Indicates the involvement in the interactions of main chain (M) or side chain (S) atoms of the protein
